# Targeting Ongoing DNA Damage in Multiple Myeloma: Effects of DNA Damage Response Inhibitors on Plasma Cell Survival

**DOI:** 10.3389/fonc.2017.00098

**Published:** 2017-05-19

**Authors:** Ana Belén Herrero, Norma Carmen Gutiérrez

**Affiliations:** ^1^Cancer Research Center-IBMCC (USAL-CSIC), Salamanca, Spain; ^2^Institute of Biomedical Research of Salamanca (IBSAL), Salamanca, Spain; ^3^Hematology Department, University Hospital of Salamanca, Salamanca, Spain

**Keywords:** DNA damage response, DNA damage, ataxia telangiectasia-mutated protein, ataxia telangiectasia and Rad3-related protein, double-strand break repair, homologous recombination, non-homologous end joining

## Abstract

Human myeloma cell lines (HMCLs) and a subset of myeloma patients with poor prognosis exhibit high levels of replication stress (RS), leading to DNA damage. In this study, we confirmed the presence of DNA double-strand breaks (DSBs) in several HMCLs by measuring γH2AX and RAD51 foci and analyzed the effect of various inhibitors of the DNA damage response on MM cell survival. Inhibition of ataxia telangiectasia and Rad3-related protein (ATR), the main kinase mediating the response to RS, using the specific inhibitor VE-821 induced more cell death in HMCLs than in control lymphoblastoid cells and U266, an HMCL with a low level of DNA damage. The absence of ATR was partially compensated by ataxia telangiectasia-mutated protein (ATM), since chemical inhibition of both kinases using VE-821 and KU-55933 significantly increased the death of MM cells with DNA damage. We found that ATM and ATR are involved in DSB repair by homologous recombination (HR) in MM. Inhibition of both kinases resulted in a stronger inhibition that may underlie cell death induction, since abolition of HR using two different inhibitors severely reduced survival of HMCLs that exhibit DNA damage. On the other hand, inhibition of the other route involved in DSB repair, non-homologous end joining (NHEJ), using the DNA-PK inhibitor NU7441, did not affect MM cell viability. Interestingly, we found that NHEJ inhibition did not increase cell death when HR was simultaneously inhibited with the RAD51 inhibitor B02, but it clearly increased the level of cell death when HR was inhibited with the MRE11 inhibitor mirin, which interferes with recombination before DNA resection takes place. Taken together, our results demonstrate for the first time that MM cells with ongoing DNA damage rely on an intact HR pathway, which thereby suggests therapeutic opportunities. We also show that inhibition of HR after the initial step of end resection might be more appropriate for inducing MM cell death, since it prevents the occurrence of a compensatory NHEJ repair mechanism. These preclinical observations provide the rationale for its clinical evaluation.

## Introduction

Multiple myeloma (MM), the second most common hematological malignancy, arises from the abnormal clonal proliferation of malignant plasma cells (1, 2). Current therapies have significantly improved survival of MM patients. On the one hand, high-dose melphalan followed by autologous hematopoietic cell transplant has become a standard of care for young patients after bortezomib-based induction regimens. On the other hand, the introduction of novel agents, particularly bortezomib combined with alkylating drugs and prednisone, or lenalidomide plus dexamethasone has also improved the outcome of patients who are ineligible for high-dose therapy (3, 4). Nevertheless, MM remains an incurable disease, and new therapeutic strategies are still needed. A prominent feature of MM cells is their genome instability, whose underlying molecular basis is not fully understood. Recently, it has been proposed that high levels of ongoing intrinsic DNA damage and deregulated double-strand break (DSB) repair influence the acquisition of genomic changes (5–8).

Double-strand breaks pose a serious threat to cell viability and genome stability if left unrepaired or if they are repaired incorrectly (9). These DNA lesions can be generated exogenously, by exposure to a variety of genotoxic agents, or endogenously, due to various factors that slow down or stall replication forks, a phenomenon known as replication stress (RS). Sources of RS include fragile sites, replication–transcription complex collision, secondary DNA structures, and oncogenic stress (10, 11). To limit the impact of DSBs, cells have evolved the DNA damage response (DDR), a signal transduction cascade that coordinates the signaling and repair of these genomic lesions (9). Ataxia telangiectasia-mutated protein (ATM) and ataxia telangiectasia and Rad3-related protein (ATR) are central components of the DDR. ATM is a serine–threonine kinase that phosphorylates several key proteins, leading to cell cycle arrest through phosphorylation of CHK2, DNA repair, or apoptosis (12–14). It is mainly activated by DSBs, such as those induced by ionizing radiation (IR). In contrast, ATR is the key kinase in signaling the response to single-strand DNA (ssDNA), which can occur at persistent DSBs and on stalled replication forks. It is considered to be the main kinase mediating the response to RS (10).

Once DNA damage has been detected, DSBs are mainly repaired by two pathways: non-homologous end joining (NHEJ) and homologous recombination (HR). NHEJ is based on a direct ligation of the two ends of damaged DNA molecules and repairs DSBs mainly in G1 phase, although it is active throughout the cell cycle (15–17). HR promotes the recovery or repair of lesions that arise during replication and has a less important role in the repair of non-replication-associated DSBs (18). The pathway starts with the 5′-to-3′ resection of DNA ends, which is initiated by the endonuclease MRE11. The generated 3′-ssDNA overhangs bind to replication protein A, which is then exchanged for RAD51, the recombinase involved in the search for homology in the sister chromatid, thereby allowing subsequent repair (19).

High levels of RS leading to DNA damage have recently been described in a subset of MM (8). These patients, who overexpressed genes belonging to a chromosomal instability and DNA damage signature, displayed poor prognosis. On the other hand, several inhibitors of proteins involved in the DDR have been developed and some of them are either close to or are already being clinically trialed (20, 21). However, the effect of these types of compounds on MM cell viability has not been investigated in depth. In this study, we confirmed the presence of DSBs in several human myeloma cell lines (HMCLs) and evaluated cell survival after inhibition of the DDR. Our results show that MM with ongoing endogenous DNA damage depends on ATR over ATM and on HR over NHEJ, providing evidence of the presence of RS in these cells. However, the absence of ATR was partially compensated by ATM, and NHEJ was activated when HR was inhibited before DNA resection. These findings suggest that multiple inhibition of the DDR or inhibition of HR after DNA resection could extend the therapeutic opportunities. The implications of our findings for the treatment of the disease are discussed.

## Materials and Methods

### Cells and Culture Conditions

The HMCL MM1S was acquired from American Type Culture Collection, and JJN3, RPMI-8226, U266, and OPM2 were obtained from Deutsche Sammlung von Mikroorganismen und Zellkulturen. LINF903, an Epstein–Barr virus-transformed B-cell line established from a healthy individual, was obtained from the National DNA Bank of the University of Salamanca, Spain. MM and LINF903 cell lines were cultured in RPMI 1640–l-glutamine medium (Sigma-Aldrich, St. Louis, MO, USA) supplemented with 10% of fetal bovine serum (Sigma-Aldrich) and antibiotics (Gibco Life Technologies, Grand Island, NY, USA). All cells were incubated at 37°C in a 5% CO_2_ atmosphere. The presence of mycoplasma was routinely checked with a MycoAlert kit (Lonza, Basel, Switzerland) and only mycoplasma-free cells were used in the experiments.

### Immunofluorescence Staining

Cells (50,000) were mounted on glass slides by cytospinning for 10 min at 1,000 rpm. Cells were fixed in 2% paraformaldehyde for 20 min, permeabilized in 0.2% Triton X-100 in PBS for 10 min, blocked in 3% BSA in PBS for 30 min and incubated with anti-γ-H2AX (mouse, clone JBW301, Millipore) or anti-Rad51 (Ab-1, rabbit, Millipore) at 1:1,000 dilution for 2 h. After washing, slides were incubated with fluorescent secondary antibodies (1:1,000, Alexa Fluor 488 goat anti-mouse IgG or Alexa Fluor 568 anti-rabbit) for 1 h. Slides were mounted with ProLong Gold antifade reagent (Invitrogen, Carlsbad, CA, USA), and images were acquired using a DeltaVision system made up of a Olympus IX71 microscope, a Photometrics Coolsnap camera, and SOFWORX software. A 60× oil immersion objective was used.

### Immunoblotting

Cells were washed with PBS and resuspended in RIPA lysis buffer (Santa Cruz Biotechnology) containing protease inhibitors (Complete, Roche Applied Science, Indianapolis, IN, USA) and phosphatase inhibitors (Santa Cruz Biotechnology). Protein concentration was measured using the Bradford assay (BioRad, Hercules, CA, USA). Protein samples (20 μg/lane) were subjected to SDS-PAGE and transferred to PVDF membrane (BioRad). After blocking, membranes were incubated with anti-human antibodies. The following primary antibodies were used: anti-p-ATM (pSer^1981^, mouse, clone 10H11.E12, Santa Cruz Biotechnology), anti-ATM (rabbit, clone D2E2, Cell Signaling, Danvers, MA, USA), anti-p-ATR (Ser428, rabbit, Cell Signaling), anti-ATR (goat, Santa Cruz Biotechnology) anti-tubulin (rabbit, Abcam, Cambridge, UK). Horseradish peroxidase-linked donkey anti-rabbit, anti-mouse, or anti-goat antibodies (Santa Cruz Biotechnology) were used as secondary antibodies at 1:10,000 dilution. Immunoblots were incubated for 1 h at RT and developed using enhanced chemiluminescence Western blotting detection reagents (Amersham Biosciences, Piscataway, NJ, USA).

### Reagents

KU-55933 and VE-821 were obtained from MedChemtronica (Stockholm, Sweden). Mirin was purchased from Sigma and NU7471 from Santa Cruz Biotechnology.

### Cell Apoptosis Assays

Apoptosis was measured using Annexin V–fluorescein isothiocyanate/propidium iodide (Annexin V-FITC/PI) double staining (Immunostep, Spain) according to the manufacturer’s guidelines.

### Cell Cycle Analysis

Cells were washed in PBS and fixed in 70% ethanol for later use. Cells were rehydrated with PBS, resuspended in 500 µl of PI/RNase staining solution (Immunostep), and incubated for 20 min at RT in the dark. Samples were analyzed using a FACSCalibur flow cytometer.

### HR Functional Assay

To determine the *in vivo* levels of HR, a reporter plasmid (22) was integrated into the genome of JJN3 and U266 cell lines as follows: cell lines were transfected with 1 µg of the HR reporter plasmid linearized by digestion with *Nhe*I. Amaxa Cell Line Nucleofector Kit V and an Amaxa Nucleofector device (Lonza, Allendale, NJ, USA) were used with programs X-005, for the U266 cell line, and T-016 for the JJN3 cell line. Three days after transfection G418 was added at 500 µg/ml. Medium containing G418 was changed every 3 days. Stable pools were obtained after 3 weeks of selection and were named U266-HR and JJN3-HR. In the chromosomally integrated reporter cassette, a unique DSB can be introduced by the rare-cutting endonuclease *I-Sce*I. Upon induction of a DSB, a functional GFP gene can be reconstituted by gene conversion, the predominant HR repair pathway in mammalian cells (22). To evaluate HR efficiency, 10^6^ cells per transfection were cotransfected with 5 µg of an *I-Sce*I-expressing plasmid together with 0.5 µg of pDsRed-N1 to normalize measurements with respect to the transfection efficiency and were incubated in the presence or absence of various DDR inhibitors. Live cells were selected by FSC/SSC gating, and live GFP+ and DsRed+ cells were quantified by flow cytometry. HR efficiency was calculated as the ratio of GFP+ to DsRed+ cells.

## Results

### HMCLs Exhibiting DNA Damage Are Hypersensitive to a Combination of ATM and ATR Inhibitors

**Figure 1:**
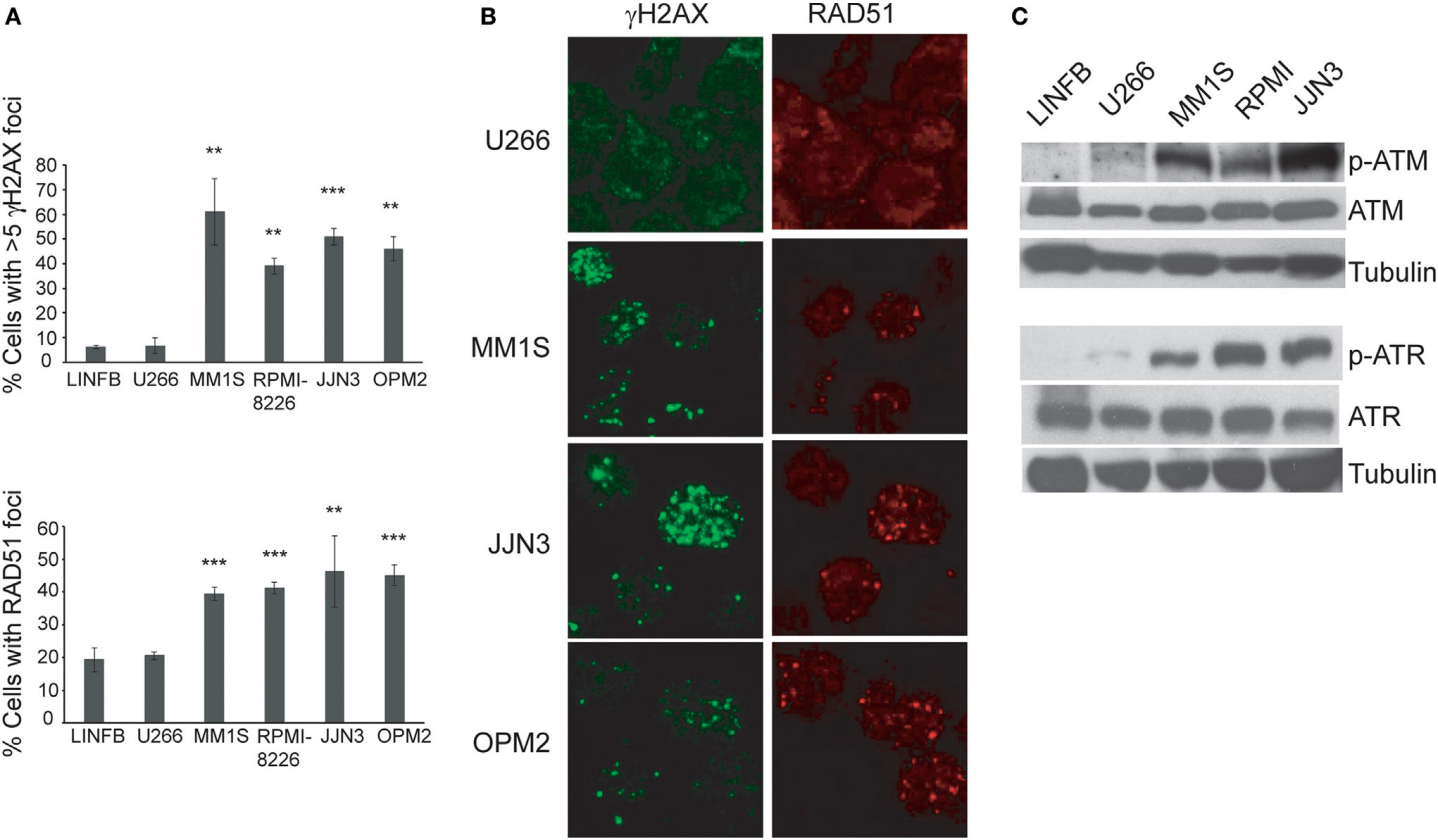
**Several human myeloma cell lines exhibit a high level of endogenous DNA damage**. **(A)** Percentage of cells with γH2AX or RAD51 foci in the indicated cell lines. Data are the mean of three independent experiments. At least 75 cells per experiment were counted. Error bars correspond to the SD (***p < 0.001 and **p < 0.01 compared with LINFB cells, Student’s *t*-test). **(B)** Representative immunofluorescence images of cells stained with anti-γH2AX or anti-RAD51 antibodies. **(C)** Western blot of kinase proteins involved in the DNA damage checkpoint.

**Figure 2:**
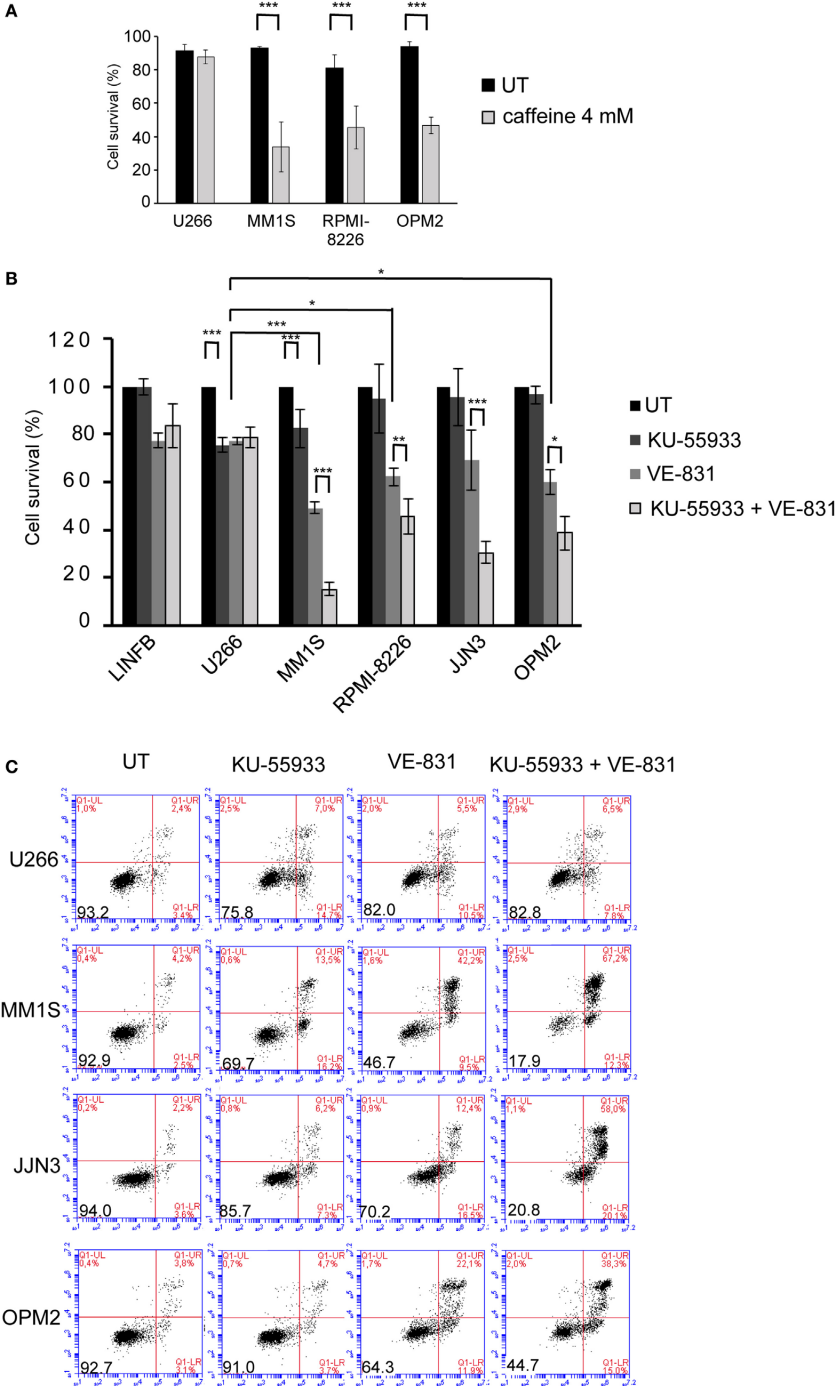
**Multiple myeloma cells with ongoing DNA damage are hypersensitive to the simultaneous inhibition of ataxia telangiectasia-mutated protein and ataxia telangiectasia and Rad3-related protein kinases**. **(A)** Cell survival after 48 h of treatment with 4 mM caffeine determined by Annexin V/propidium iodide (Annexin V/PI) staining. **(B)** Cell survival after 72 h in the absence (untreated = UT) or the presence of 10 µM KU-55933, 5 µM VE-821, or both. Cell viability in the absence of treatment (only DMSO) was taken as 100%, and values obtained for all other conditions were normalized with respect to the UT value. **(C)** Examples of flow cytometry dot plots of cells stained with Annexin V/PI after 72 h of treatment with the indicated chemical inhibitors. Data are the mean of at least three independent experiments (***p < 0.001, **p < 0.01, and *p < 0.05).

Recent reports have shown ongoing constitutive DNA damage in several HMCLs and in plasma cells isolated from patients (6, 8). To corroborate these findings we quantified γ-H2AX and Rad51 foci, markers of DSBs, in HMCLs and in LINF903, a lymphoblastoid B cell line obtained from normal lymphocytes (7). HMCLs exhibited a higher percentage of cells with γ-H2AX and Rad51 foci than control lymphoblastoid cells, with the exception of U266 (Figures 1A,B), in agreement with recent published data (8). Activation of the DDR was detected in HMCLs with ongoing DNA damage by the presence of p-ATM and p-ATR (Figure 1C). The confirmation of high levels of DNA damage in most of the HMCLs prompted us to investigate the effect of various inhibitors of proteins involved in the DDR on MM cell viability. First, we analyzed the sensitivity of MM cell lines exhibiting low (U266) or high levels of DNA damage to caffeine, a well-known inhibitor of both ATM and ATR kinases. MM1S, RPMI-8226, and OPM2 were found to be more sensitive to the drug than U266, as revealed by Annexin V-FITC/PI staining (Figure 2A). Specific inhibitors were then used to determine whether cell death induced by caffeine in HMCLs was due to inhibition of ATM, ATR, or both kinases. Inhibition of ATM with 10 µM KU-55933 resulted in a small reduction in cell survival in U266 and MM1S cells relative to untreated cells, whereas no significant effect was detected in any other HMCLs (Figure 2B). On the other hand, pharmacological inhibition of ATR with 5 µM of the specific inhibitor VE-821 (23) triggered a stronger apoptotic response in MM1S, RPMI-8226, and OPM2 than in LINF903 and U266 cell lines, in agreement with a recent report (8). Interestingly, inhibition of both kinases using a combination of the two drugs significantly increased cell death caused by ATR inhibition only in cells with high endogenous DNA damage (Figures 2B,C). These results indicated that ATM compensates for the absence of ATR, since the level of cell death caused by both inhibitors was higher than that observed with the individual inhibitors.

### ATM and ATR Participate in DSB Repair by HR in MM, and Inhibition of Both Kinases Produces a Stronger Inhibitory Effect

**Figure 3:**
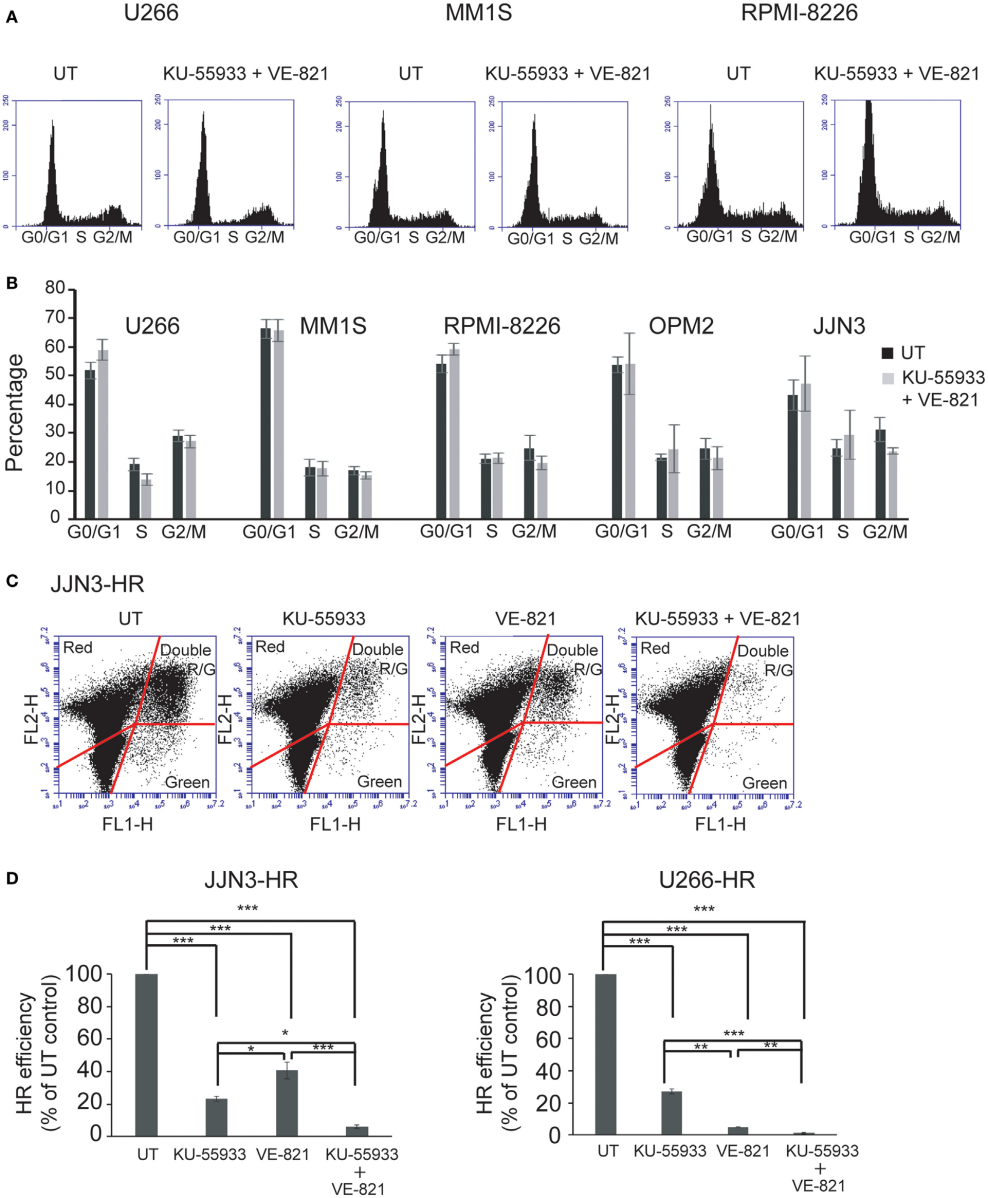
**Inhibition of ataxia telangiectasia-mutated protein and ataxia telangiectasia and Rad3-related protein does not affect cell cycle profiles of multiple myeloma cells but greatly reduces the efficiency of homologous recombination (HR) by gene conversion**. **(A)** Cell cycle profiles of the indicated human myeloma cell lines untreated (UT) or treated with 10 µM KU-55933 and 5 µM VE-821. Cells were collected after 30 h of treatment and stained with propidium iodide. **(B)** Distribution of cells in G1, S, and G2/M phases of the cell cycle. **(C)** GFP+ and DsRed+ cells after 30 h of transfection with 5 µg of I-*Sce*I endonuclease-expressing plasmid and 0.5 µg pf pDSRed2-N1. **(D)** HR efficiency was calculated as the ratio of GFP+ to DsRed+ cells. The ratio in UT cells was taken to be 100% and in all other situations calculated as the percentage of UT controls. Data are the mean of three independent experiments. Error bars represent the SD (***p < 0.001, **p < 0.01, and *p < 0.05).

Ataxia telangiectasia-mutated protein and ATR kinases are mainly known for phosphorylating their substrates CHK2 and CHK1 leading to cell cycle arrest in G1 or G2 (12, 24–26). However, even though many HMCLs exhibited endogenous DNA damage we found that cell cycle profiles did not change significantly after treatment with the combination of ATM and ATR checkpoint inhibitors (Figures 3A,B). This result suggests that ATM and ATR may carry out other functions in cells with DNA damage that make them dependent on the presence of both kinases. It has previously been shown that unrepaired DSBs can lead to cell death (27) and also that ATM is important for DSB repair by HR, since its downregulation reduces HR efficiency (14). Based on these findings, we hypothesized that inhibition of both kinases might strongly affect DSB repair by HR, leading to the death of MM cells with accumulated DNA damage. To analyze whether inhibition of ATM, ATR, or both caused defects in recombination, we took advantage of an HR substrate that was integrated within the chromatin of the MM cell lines JJN3 and U266. DSBs were then generated by transfection of the resulting JJN3-HR and U266-HR cells with an I-*Sce*I endonuclease-expressing plasmid, and HR efficiency in the presence or absence of the kinase inhibitors was estimated. We found that ATM inhibition by treatment with KU-55933 (10 µM) strongly reduced HR efficiency in JJN3-HR and U266-HR (Figures 3C,D), although cells were still able to perform HR to some extent. ATR inhibition by VE-821 also resulted in a notable reduction in HR that was lower than that induced by ATM inhibition in JJN3-HR, but higher in U266-HR. A stronger suppressive effect on HR was observed when both kinases were simultaneously inactivated, indicating that ATM partially compensates for the lack of ATR and *vice versa*.

### Total Inhibition of Recombination Severely Affects Survival of MM Cells with Ongoing DNA Damage

**Figure 4:**
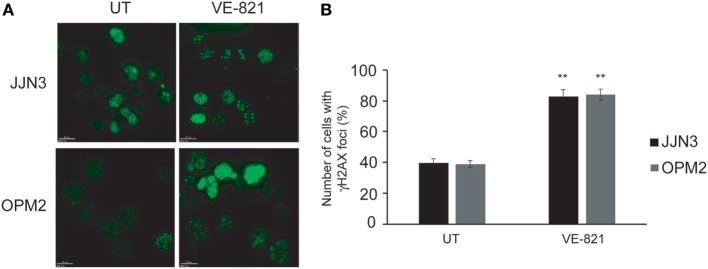
**Inhibition of ataxia telangiectasia and Rad3-related protein results in the accumulation of DNA double-strand breaks**. **(A)** γH2AX foci in untreated (UT) cells and after 48 h of treatment with 5 µM VE-821. **(B)** Quantification of cells with foci. A total of 100 cells per cell line and condition were counted. Data are the mean of three independent experiments. Error bars represent the SD (**p < 0.01 compared with UT cells).

Based on the results reported above we speculated that sensitivity to ATR inhibition is caused by an additional role of this protein to that performed in DSB repair. Cell death induced by the chemical inhibition of both kinases could be caused by the individual effect of ATR inhibition along with the more marked decrease in HR capability. It has previously been shown that inhibition of ATR in conditions of RS leads to replication fork collapse and the accumulation of DSBs (28, 29), so we hypothesized that such accumulation, together with defects in DSB repair, might underlie hypersensitivity to ATR inhibition in MM. To explore this possibility, cells were treated with VE-821, and the presence of DSBs was analyzed by monitoring γH2AX foci. As shown in Figures 4A,B, treatment of MM cells with VE-821 clearly increased the number of cells with γH2AX foci relative to untreated controls. Number of γH2AX foci per cell also increased and in several cells became uncountable.

**Figure 5:**
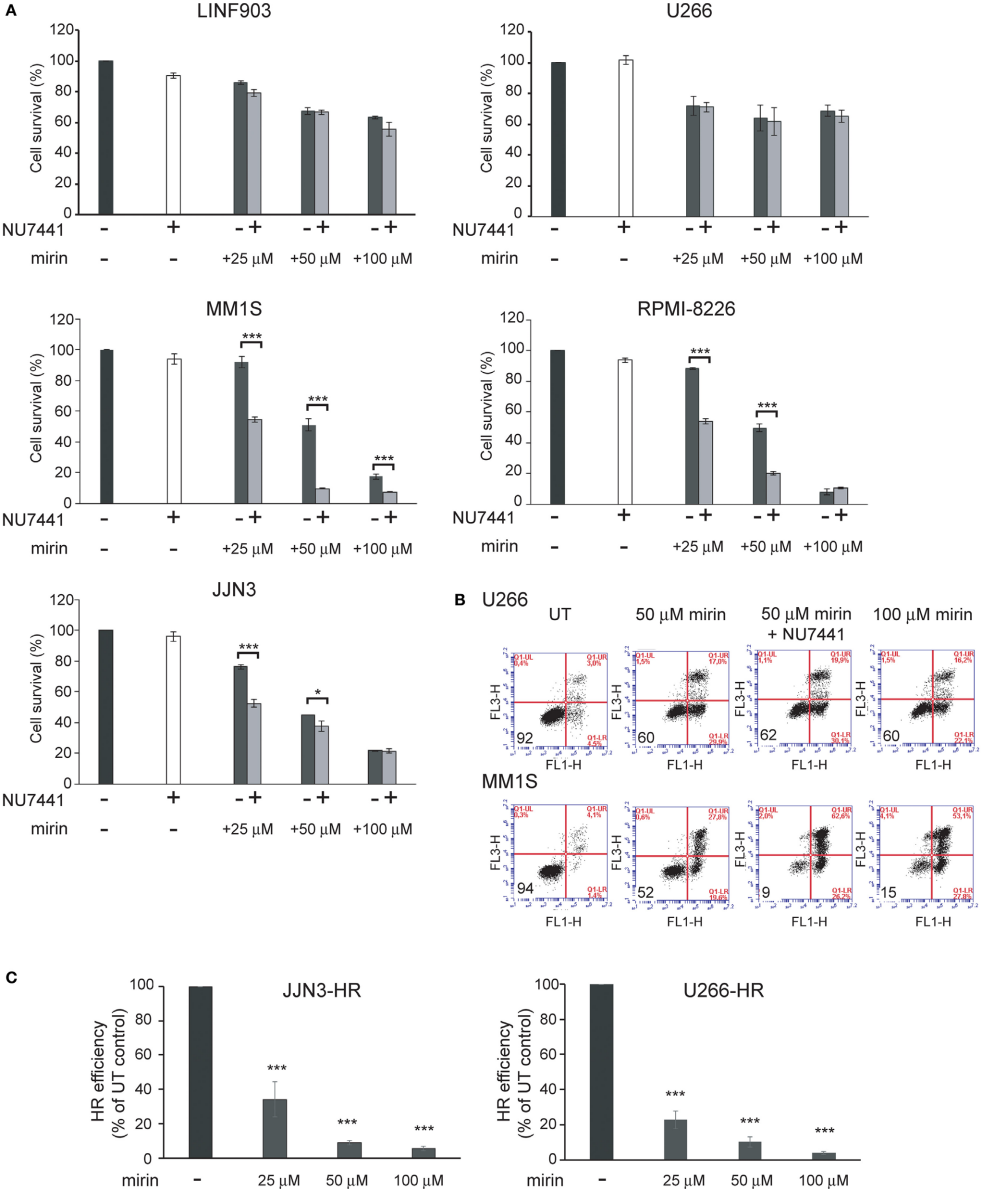
**Mirin induces cell death of multiple myeloma cells with endogenous DNA damage and reduces homologous recombination (HR) efficiency in a dose-dependent manner**. **(A)** Cell survival determined by Annexin/propidium iodide (Annexin/PI) staining after 72 h of treatment with the indicated compounds. **(B)** Representative flow cytometry dot plots of cells stained with Annexin/PI. **(C)** HR efficiency in the indicated conditions. Data are the mean of three independent experiments. Error bars correspond to the SD (***p < 0.001 and *p < 0.05).

To explore whether the strong inhibition of HR causes the death of MM cells with ongoing DNA damage we used mirin, an MRE11 inhibitor that abolishes HR by impeding DSB resection (30). We also explored the contribution of the other main pathway involved in DSB repair, NHEJ, to the survival of MM cells exhibiting DNA damage. For this purpose, we used NU7441, a NHEJ inhibitor that targets DNA-PK (31). We found that treatment for 72 h with mirin reduced survival of MM1S, RPMI-8226, and JJN3 in a dose-dependent manner, whereas survival of LIN903 and U266 cells was higher, as expected, and remained similar at the different doses of mirin employed (Figures 4A,B). On the other hand, inhibition of NHEJ with concentrations of NU7441 up to 40 µM did not affect survival of any of the cell lines analyzed, but clearly decreased survival of HMCLs with a high level of ongoing DNA damage when treated with 25 and 50 µM mirin. Based on these results we concluded that mirin at 25 or 50 µM might not completely inhibit HR, and, if so, cells might survive HR inhibition by the compensatory NHEJ pathway. To explore this hypothesis, JJN3-HR and U266-HR cells carrying the HR reported cassette were treated with different doses of mirin, and HR efficiency was analyzed after 30 h of culture (Figure 5C). We found that the recombination efficiency of live cells decreased with increasing doses of mirin, from a partial loss of efficiency at 25 µM to a nearly total loss at 100 µM. These results indicated that HMCLs with ongoing DNA damage are hypersensitive to HR inhibition and also that defects in DNA resection induced by mirin can be compensated by NHEJ, a mechanism able to rejoin broken molecules before total resection occurs (32). Importantly, the reduction in HR was very similar in JJN3-HR and U266-HR (Figure 5C), which demonstrated that U266 was more resistant to mirin because it suffered less endogenous DNA damage, and not because mirin was not active in these cells.

**Figure 6:**
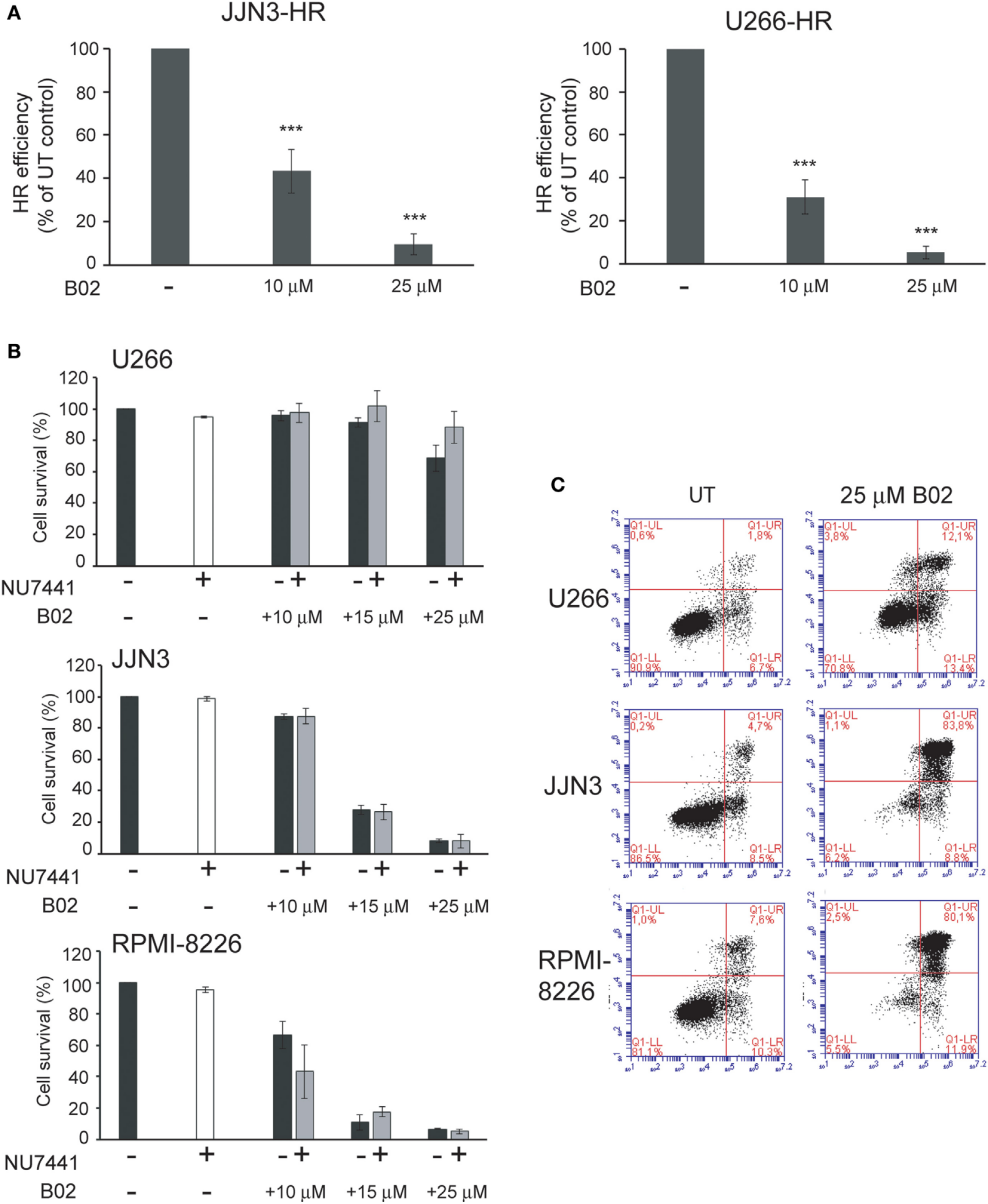
**B02 reduces homologous recombination (HR) efficiency and induces multiple myeloma cell death**. **(A)** Efficiency of HR after treatment with B02. **(B)** Cell survival determined by Annexin–fluorescein isothiocyanate/propidium iodide staining of human myeloma cell lines (HMCLs) treated for 72 h with the indicated inhibitors. A total of 10,000 events are shown. **(C)** Representative dot plots of the indicated HMCLs. Data are the mean of three independent experiments. Error bars represent the SD (***p < 0.001).

**Figure 7:**
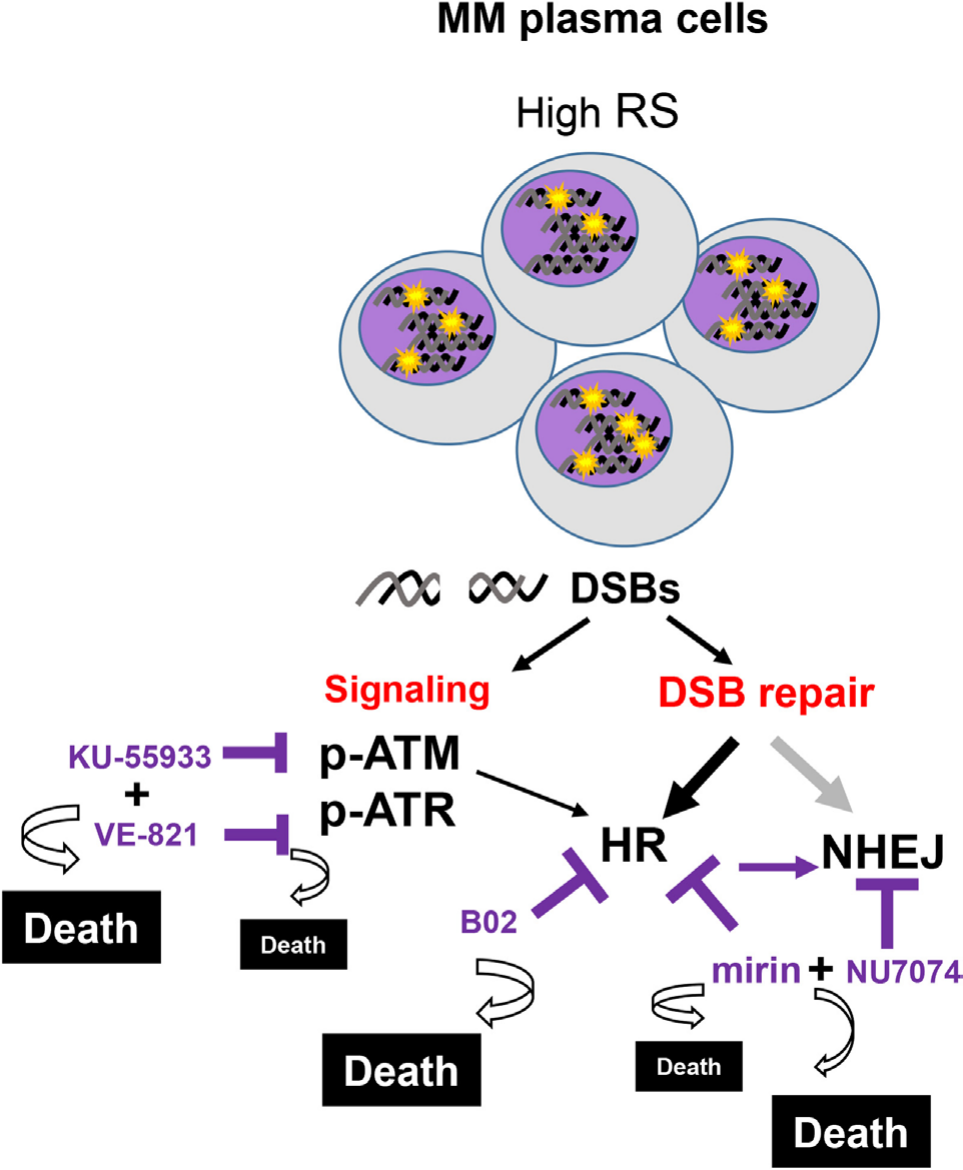
**Several multiple myeloma patients present PCs with high levels of replication stress (RS), leading to double-strand breaks (DSBs)**. These cells activate the DNA damage response that coordinates the signaling and the repair of the lesions. Replication-associated DSBs are mainly repaired by homologous recombination (HR). Targeting HR by dual inhibition of ataxia telangiectasia-mutated protein and ataxia telangiectasia and Rad3-related protein or by using the HR inhibitor B02 would be particularly toxic for these cells. High levels of cell death will also be achieved using mirin and NU7471, since the last inhibits the compensatory non-homologous end joining mechanisms. In contrast, normal cells with minimal levels of RS are expected to survive these treatments.

To confirm the dependency of MM cells on HR we used B02, a recently discovered inhibitor of Rad51 (33). HMCLs were treated with different doses of B02, and HR efficiency and cell viability were analyzed. As expected, B02 clearly inhibited HR in both U266-HR and JJN3-HR reporter cell lines (Figure 6A), although inhibition of HR resulted in more cell death in JJN3 and RPMI-8226 than in U266 (Figures 6B,C), further confirming that MM cells with ongoing DNA damage are hypersensitive to HR inhibition. We found that simultaneous inhibition of HR using B02 and NHEJ with NU7441 did not increase cell death induced by B02 alone (Figure 6B). This result clearly indicated that endogenous DNA damage in MM is mainly repaired by HR and that if this pathway is inhibited after DNA resection occurs the break can no longer be repaired by a compensatory NHEJ mechanism. An illustration summarizing all the results is shown in Figure 7.

## Discussion

High levels of DNA damage can make tumor cells dependent on a proper DDR and represent a vulnerability of cancer cells that could be targeted therapeutically (34). In this study, we show that inhibition of the DDR is particularly toxic for MM cells exhibiting high levels of DSBs. Specifically, MM cells were found to be hypersensitive to agents that inhibit DSB repair by HR, a pathway upregulated in MM and important for the resolution of breaks associated with RS.

Recently, Cottini et al. showed that several HMCLs suffer RS, which is induced, at least in part, by the overexpression of MYC. In fact, silencing this oncogene in HMCLs exhibiting DNA damage and MYC overexpression reduced the number of lesions. On the other hand, its overexpression in U266, an HMCL with low levels of DNA damage and no MYC overexpression, triggered an increase in DSBs (8). The authors also found that ATR, the main kinase involved in the RS response, promoted survival of MYC-overexpressing cells. Here, we confirm the presence of high levels of DNA damage in several HMCLs, with the exception of U266, whose level of endogenous DNA damage was similar to that found in normal lymphoblastoid B cells, which were used in this work as healthy controls. We also confirmed that HMCLs exhibiting DNA damage rely on activated ATR for survival. Moreover, we observed that these cells were more sensitive to ATR than to ATM inhibition, probably because the first induces replication fork collapse (28, 29), leading to the accumulation of DSBs (Figure 4). However, we found that the absence of ATR was compensated by ATM, since cell death induced by the inhibition of both kinases was more extensive than that obtained using single kinase inhibitors. It is especially noteworthy that the lethal effects of ATM and ATR inhibitors were found only in cells with extensive endogenous DNA damage, which bolsters the therapeutic opportunities. ATR and ATM transduction pathways were initially considered as two parallel pathways: IR induced the ATM pathway, whereas RS activated the ATR pathway. However, the regulation of ATM and ATR was later shown to be mutually dependent in response to various DNA-damaging conditions like UV light, IR, and replication stalling (35–39). Thus, ATM has been demonstrated to play a role in maintaining fragile site stability, which is revealed only in the absence of ATR (40). Our results showing an increase in MM cell death with the combination of the two kinase inhibitors indicate the importance of both proteins for coping with endogenous DNA damage in MM and suggest that a double therapy could be more efficient for killing malignant cells. In this regard, it has been reported that in *ATM*-defective chronic lymphocytic leukemia cells, inhibition of ATR signaling by AZD6738 leads to an accumulation of unrepaired DNA damage that results in cell death by mitotic catastrophe (41). Dual inhibition of ATR and ATM has also been shown to potentiate the activity of the DSB-inducing drugs trabectedin and lurbinectedin by perturbing the DDR and HR repair (42).

We show for the first time that ATM and ATR are involved in DSB repair by HR in MM and also that inhibition of both kinases completely abolishes HR efficiency. These results, obtained using an integrated-GFP-based DNA repair reporter substrate, support a previously described model in which ATM and ATR collaborate to maintain the activity of CtIP for efficient DNA end resection during HR (43). We found that ATM/ATR inhibition did not affect cell cycle distribution of HMCLs with high endogenous DNA damage (Figures 3A,B), which led us to hypothesize that the effect on HR, together with the accumulation of DSBs due to ATR inhibition, might underlie cell death induction. This hypothesis was supported by the finding that abolition of HR using the MRE11 inhibitor mirin, or the RAD51 inhibitor B02, severely decreased survival of HMCLs exhibiting high levels of endogenous DNA damage. Here, we show that inhibition of the other route involved in DSB repair, NHEJ, using the DNA-PK inhibitor NU7441 did not affect MM cell viability, which clearly demonstrated that endogenous DNA damage in MM is repaired by HR. We found that NHEJ inhibition did not increase cell death when HR was simultaneously inhibited with B02, but did clearly enhance cell death when HR was inhibited with mirin, which interferes with recombination before DNA resection occurs. These results are consistent with previous observations by Shibata et al., who showed that inhibition of the endonuclease activity of MRE11 promoted NHEJ (32). They are also consistent with the notion that 3′-ssDNA ends generated after DNA resection as substrates for HR can no longer be channeled toward NHEJ (18). Importantly, our results indicate that inhibition of HR after the initial step of end resection might be more appropriate for inducing MM cell death, since it prevents the occurrence of a compensatory NHEJ repair mechanism.

Many studies have shown that combinations of genotoxic agents with inhibitors of the DDR produce greater cell death of tumor cells compared with single agents (21, 44–46). In MM, the cytotoxic effects of bendamustine, melphalan, and doxorubicin on p53-deficient cells are enhanced by AZD7762, a CHK1/CHK2 inhibitor (47). In addition, inhibition of RAD51 has been found to sensitize MM cells to IR and to the DSB-inducing drug doxorubicin (48). However, the present study is the first to indicate that the single-agent activity of HR inhibitors induces apoptosis of MM cells with intrinsic DNA damage, providing a therapeutic window. It is important to note that partial inhibition of HR did not result in complete myeloma cell death at the times assayed, probably because residual traces of activity may be enough to ensure repair of endogenous DSBs. However, doses that completely abolished HR, such as the combination of ATM and ATR inhibitors, or concentrations of B02 greater than 15 µM drove most of the MM cells with intrinsic DNA damage to apoptosis. It is possible that the high doses of HR inhibitors needed to induce complete MM cell death could have a toxic effect, especially over prolonged periods, but maybe one that is less severe than that induced by combinations of DDR inhibitors with genotoxic agents, since the latter induce DNA damage and are also toxic to non-tumor cells. Nevertheless, specific (or temporary) inhibition of HR could be efficient not only for reducing MM cell survival but also for mitigating genomic instability and disease progression, given that we and others have demonstrated a high level of RAD51 expression and increased HR that lead to the genome instability characteristic of the disease (5, 7). Taken together, our findings suggest that HR inhibition could be a promising target for the treatment of MM. Therapeutic outcomes and toxicity profiles of the different inhibitors used as single agents or in combinations need to be tested in clinical trials.

## Author Contributions

AH conceived and designed the work, performed the experiments, wrote the manuscript, and approved the final version of the manuscript. NG contributed to design the work, revised it critically for important intellectual content, ensured that any part of the work was appropriately investigated and resolved, corrected the manuscript, and approved the final version to be published.

## Conflict of Interest Statement

The authors declare that the research was conducted in the absence of any commercial or financial relationships that could be construed as a potential conflict of interest. The reviewer, ES, and handling editor declared their shared affiliation, and the handling editor states that the process nevertheless met the standards of a fair and objective review.
